# Strenuous Exercise-Induced Tremendously Elevated Transaminases Levels in a Healthy Adult: A Diagnostic Dilemma

**DOI:** 10.1155/2021/6653266

**Published:** 2021-03-10

**Authors:** Prabin Khatri, Aryan Neupane, Suman Raj Sapkota, Bibhav Bashyal, Dipesh Sharma, Ashmita Chhetri, K. C. Chirag, Ashish Banjade, Priyanka Sapkota, Subarna. Bhandari

**Affiliations:** Universal College of Medical Sciences & Teaching Hospital, Bhairahawa, Nepal

## Abstract

The liver function test (LFT) is a commonly performed test in clinical practice in order to assess well-being of the liver; however, derangement in liver enzymes, however, may not necessarily imply an underlying liver pathology. The standard liver function test measures alanine aminotransferase (ALT), aspartate aminotransferase (AST), alanine phosphatase (ALP), bilirubin levels (total, direct, and indirect), proteins (total protein and albumin), and PT-INR (prothrombin time and international normalized ratio). In addition to common causes, liver enzyme levels can also be elevated due to extrahepatic causes, such as muscular injury can elevate transaminases levels. Here in, we present a case of an asymptomatic healthy male who was doing vigorous exercise and presented with reports of elevated transaminase levels. During evaluation of the case, most of his reports came to be within normal range. Additionally, when reevaluated after discontinuation of vigorous exercise, 3 weeks later and then a month later, his liver enzyme levels were observed to be within normal range. Hence, we suspect that muscle damage-induced transaminitis might not have been considered in the differential diagnosis during the evaluation of a patient with raised transaminases levels and also suggest that it should be kept as a differential in the given scenario.

## 1. Introduction

Liver function test (LFT) is a commonly performed test in clinical practice. Assessment of liver enzymes is an important component of the liver function test. Derangement in the liver enzymes, however, may not necessarily imply an underlying liver pathology [1]. Some of the common causes of deranged liver enzymes are liver injury due to alcohol, nonalcoholic fatty liver disease, hemochromatosis, hepatitis B, hepatitis C, illegal drugs, dietary supplements, and over-the-counter and prescription medications, including many psychiatric drugs [2, 3]. In addition, alpha-1-antitrypsin deficiency, autoimmune hepatitis, and Wilson disease are considered to be among the rarer causes [2].

Although present in high concentrations in the liver, AST (aspartate aminotransferase) is also present in the muscle, heart, kidney, red blood cells, brain, and small bowel, whereas ALT (alanine aminotransferase) is present in the liver, muscle, and kidney. Owing to a larger tissue mass, muscle has more AST and ALT when compared with that in the liver. Due to this reason, in muscular disorders or injury (e.g., myocardial infarction, surgery, and vigorous exercise), hemolysis, and small bowel ischemia, the transaminases levels may be elevated [4, 5]. Abnormal results in the liver function test often cause considerable anxiety in asymptomatic subjects. It may lead to visits to multiple doctors to seek advice or demand for more investigations to ascertain if there is an underlying serious liver disease [6, 7].

Here, we report a case of an asymptomatic healthy male who presented with liver function test reports with elevated transaminase levels.

## 2. Case Report

A 23-year-old male visited our outpatient department with reports of deranged liver enzymes. The AST was 410 IU/L (10–45 IU/L), and ALT was 209 IU/L (5–45 IU/L). ALP (alkaline phosphatase), GGT (gamma glutamyl transferase), and total bilirubin were within normal limits. Prothrombin time, international normalized ratio, and albumin levels were also normal.

On history taking, he was apparently asymptomatic. He explained that he had done routine investigations including liver function tests as a part of his routine checkups. He stated that he consumed alcohol, about 2–4 units/week for the last 1.5 years, with the last intake (binge drinking) about 6 days prior to the liver function test reports but did not take part in any recreational drugs and was a nonsmoker. He denied any family history of liver disease. On examination, he was averagely built, with normal vitals and without any abnormal findings in general and systemic examination.

The patient underwent baseline investigations including complete blood count, renal function test, lipid profile, liver function test, urine routine microscopy, serology for hepatitis B and C, and HIV which were all within normal limits apart from raised transaminase levels. An ultrasonographic examination of the abdomen was unremarkable.

The initial impression was transaminitis secondary to alcohol consumption. We advised the patient to refrain from alcohol intake and take adequate rest with the plan to repeat LFT after 3 weeks. As the patient was anxious about his report, he again repeated his LFTs 3 days later. This time the AST level was 580 IU/L (previously 408 IU/L), and ALT was 277 IU/L (previously 209 IU/L). On inquiry, he reported no alcohol consumption. This increasing trend of AST and ALT needed further investigations to determine the etiology. Further investigations included serology for hepatitis A and E, dengue, anti-nuclear antibody (ANA), thyroid function tests (TFTs), and serum ceruloplasmin, all of which were within normal limits, while the anti-LKM antibody and anti-Smith antibody tests were negative.

On repeated inquiry, the patient said that he was experiencing mild muscle ache which he thought was due to his vigorous workouts which he had started doing a week before doing LFT reports. This involved intensive weight-based training covering various groups of muscle for 2 hours every day for a week. This prompted us to send creatine phosphokinase (CPK) and lactate dehydrogenase (LDH). The reports came out as CPK = 21350 U/L (55–170) and LDH = 720 U/L (140–280). Then, the patient was suspected to have transaminitis due to muscle damage. He was then advised to take adequate rest and refrain from his daily workouts. The LFT, CPK, and LDH were repeated after 3 weeks which all came out to be within normal limits, thus strongly pointing towards our suspicion of muscle damage-induced transaminitis. These tests, repeated after 1 month, were also within normal limits.

## 3. Discussion

The transaminases are commonly referred to as “liver enzymes,” but this is not entirely true. It should not be forgotten that they may be elevated due to extrahepatic causes [4, 5]. The common causes of raised transaminases levels are liver injury due to alcohol, nonalcoholic fatty liver disease, hemochromatosis, hepatitis B, hepatitis C, illegal drugs, dietary supplements, and over-the-counter and prescription medications, including many psychiatric drugs. [2, 3]. Amongst the rare causes are alpha-1-antitrypsin deficiency, autoimmune hepatitis, and Wilson disease [2]. Muscular disorders or injury (e.g., myocardial infarction, surgery, and vigorous exercise), hemolysis, and small bowel ischemia are amongst the extrahepatic causes in which transaminase levels may be elevated [4, 5]. The American Gastroenterological Association (AGA) technical review states that there is diurnal variation in serum ALT, which may even vary day-to-day and may be affected by muscle injury or exercise [8]. Muscle has more AST and ALT when compared with that in the liver because of a larger tissue mass [4].

Routine blood investigations including LFTs are commonly done as part of routine checkup in our setting even in asymptomatic individuals like that of our patient. The easy availability of liver function tests has increased the pick-up rate of liver disease. A routine checkup for our patient took him down a diagnostic pathway, which included a number of investigations, both cheap and expensive.

In a research that involved healthy men who did regular moderate physical activity, not including previous weightlifting, ALT, AST, lactate dehydrogenase, CK, and myoglobin levels significantly increased after 1 hour of heavy weightlifting and remained elevated for at least 1 week. Bilirubin, GGT, and ALP levels remained within the reference range [5]. In the setting of elevated transaminase levels, physicians do not routinely order a CK level. In this case, if the CK level had not been obtained, we could have wrongly assumed that elevated transaminase levels were manifestations of liver injury and this might have led to further investigations including an invasive procedure like liver biopsy. In a case presenting with elevated transaminases, as comparable to our case, a meticulous history should also include the exercise pattern along with other relevant points.

## 4. Conclusion

We suspect that muscle damage-induced transaminitis might not have been considered in the differential diagnosis during the evaluation of a patient with raised transaminases levels and suggest that it be kept as a differential in the given scenario. This raises a strong argument for inquiring about exercise habits and the inclusion of CPK and LDH blood tests in the screening panel since this might result in the prevention of inappropriate and exorbitant investigations including invasive tests and inappropriate referrals.

## Data Availability

The data (images of the investigations) used to support the findings of this study are available from the corresponding author on reasonable request.
